# A New Method of Using Vulcanite

**Published:** 1860-07

**Authors:** P. G. C. Hunt


### A NEW METHOD OF USING VULCANITE.

BY F. G. C. HUNT.

TAKE the impression, make metallic dies and form the plate as for work in the ordinary way. After fitting the plate in the mouth get the articulation the fullness and length of the teeth, remove the wax and plate from the mouth and make the plaster articulation. If a full set, after separating the articulation and before removing the wax from the plate, take a small light pair of dividers, set them say one inch apart and with one point following the margin of the wax representing the cutting edge of the teeth, and the other point marking permanently the plaster. Thus you always have in the dividers so set a gauge for the length of any particular tooth. A convenient substitute for the dividers may be formed from a piece of wire of convenient length, one half the diameter of a common excavator, by suitably twisting its middle for a handle, and its ends being sharpened and pointing in the same direction, one or one and a half inches apart, for which idea and many others of practical utility we are indebted to Dr. W. M. Hunter, of Cincinnati.

Thus far we proceed as we do for ordinary gold work. We will now suppose the teeth ground and jointed, leaving as much space between the teeth and plate as the plate will admit of. We next mark with a sharp pointed instrument on the labial surface of the plate each point where it is necessary to place a loop for purposes hereinafter described. Then apply wax to the external or labial parts of the teeth and plate in any manner sufficient to retain the teeth in position—remove the wax from the lingual parts of the teeth and plate and mark the position on the metal where it is desirable to insert loops, remove the teeth and wax and with a small bow-drill, make holes through the plate at the several points previously determined on for the attachments, about the size of the ordinary plate punch hole, take of ordinary gold plate, cut in strips, say from a half to one line in width, being governed by the amount of room there is, under the base of the teeth, with small round nosed pliers, or a wire, bend the strip around, grasp both ends with square nosed pliers, draw the round nosed pliers, or wire, as the case may be, still grasping the square nosed pliers with the left hand, and with a hammer strike the top of the loop a sufficient blow to keep the ends from springing apart. Cut off the ends and dress down to fit the holes in the plate, after which solder on charcoal or other suitable substance without investment. Pickle, dress and polish that portion of the plate to be exposed to view. Bend and flatten the pins, arrange the teeth according to the articulation, waxing so as to cover up the loops if practicable—the loops should be placed as near the base of the teeth as possible, the rubber forming when finished a part of that general concave shape which is desirable in upper dentures and which it is not possible to obtain with the ordinary soldered work. Then with silicate of soda paint the joints to keep the rubber from forcing in where it would show after vulcanizing. Flask, vulcanize and finish up as usual. The advantages of this style of work are obvious. With this you have work as cleanly as the continuous gum, decidedly more so than the very best single gum or block work soldered in the usual way; again, it is very much stronger, less liable to breakage both in and out of the mouth as the rubber gives *a perfect base* and support for the teeth to set upon. By this method *there is no springing of plates.* As your plates fit the mouth when the articulation was taken, so will be the fit when the case is completed.

On the labial edge of the upper plate the rubber may be allowed to project beyond the edge if desirable, and it will be found in many cases exceedingly satisfactory to do so, and allow the rubber to be of considerable thickness near the alœ of the nose where the loss of the cuspidati may leave a want of support to the soft parts adjacent, and which in this manner can be readily corrected. If the rubber extends upward so far as to irritate the muscular structure a few minutes will be sufficient to make the necessary alterations. In all such cases where we have control of our patients we place the denture in the mouth before finally polishing so as to determine as accurately as possible the limit to which extension upward may be carried.

The neatest work on this principle is made by carving blocks, giving to the lingual surface that regular concave form which is desirable. In this no platium pins or loops are necessary, but that half of the matrix on which the blocks are carved, large metallic pins are so arranged as to be hid from view in the tooth body. Different sized pins may be used as large as the nature of the case will admit. In short, we make holes in the block similar to those in pivot teeth, where there is not sufficient room in the block above the tooth (or below if an under,) to allow the pins to run into the body of the teeth. After burning, grinding and fitting, get the position of the holes in the blocks relative to the plate, and drill through the plate as before, and instead of loops solder gold wire of suitable size and length, say a very little shorter than the depth of the hole in the blocks and two thirds the diameter thereof; the wire should have a screw thread cut on it or that which is just as good and more expeditious, barb or cut with a sharp knife. At this point of the manipulation if it is desired that the rubber should extend beyond the labial or buccal edge of the metallic plate place as many loops at different points as are sufficient to retain it with firmness, after which polish the plate, wax and proceed as before described. If you desire no rubber beyond the blocks the roughness of the holes in the same and the barbed points on the gold wire when properly packed and vulcanized will give ample strength and firmness to the case, and if care has been used in the entire manipulation you will have when finished but a thin line of rubber exposed to view.

In partial cases, if of gold base, we solder on loops as before for the retention of the teeth, and if there are to be *any clasps* make them of rubber, uniting them, as the teeth, with loops. If the ordinary plate teeth are used it is frequently necessary to back them, to give better retaining points for the rubber. If blocks are to be burned insert loops of platinum plate in the shape of the letter U in place of the platinum wire pins. In consequence of the affinity of the sulphur in the vulcanite for silver, plates of that metal should not be used.

Since preparing the above I find in the May number of the *Vulcanite*, a new quarterly journal of New York, published by B. W. Franklin, a notice of a patent obtained by Drs. A. M. Asay and J. T. Asay, of Philadelphia, for the same method of mounting work as is here set forth. This method has been used by me since October, 1859, during which time I may have been trespassing upon the patented rights of Drs. Asays. The reception of the *Vulcanite* was the first intimation I had that any one was using the vulcanite in this way, excepting to those whom I have heretofore explained my method of manipulation.

